# Endurance-dependent urinary extracellular vesicle signature: shape, metabolic miRNAs, and purine content distinguish triathletes from inactive people

**DOI:** 10.1007/s00424-023-02815-x

**Published:** 2023-05-08

**Authors:** Tiziana Pietrangelo, Carmen Santangelo, Danilo Bondi, Paolo Cocci, Raffaela Piccinelli, Francesco Piacenza, Enrica Rosato, S. N. Afifa Azman, Enrico Binetti, Marco Farina, Marcello Locatelli, Virgilio Brunetti, Cinzia Le Donne, Lorenzo Marramiero, Ester Sara Di Filippo, Vittore Verratti, Stefania Fulle, Valentina Scollo, Francesco Palermo

**Affiliations:** 1 Department of Neuroscience, Imaging and Clinical Sciences, University “G. d’Annunzio” Chieti-Pescara, Chieti, Italy; 2 School of Biosciences and Veterinary Medicine, University of Camerino, Camerino, Italy; 3 Research Centre for Food and Nutrition, Council for Agricultural Research and Economics, Roma, Italy; 4 IRCCS-Istituto Nazionale di Riposo e Cura per Anziani, Polo Scientifico e Tecnologico, Centro di Tecnologie Avanzate nell’Invecchiamento, Ancona, Italy; 5 Department of Pharmacy, University “G. d’Annunzio” Chieti-Pescara, Chieti, Italy; 6 Department of Information Engineering, Polytechnic University of Marche, Ancona, Italy; 7 Center for Biomolecular Nanotechnologies, Italian Institute of Technology, Lecce, Italy; 8 Institute for Microelectronics and Microsystems, National Research Council of Italy, Lecce, Italy; 9 Department of Psychological, Health and Territorial Sciences, University “G. d’Annunzio” Chieti-Pescara, Chieti, Italy

**Keywords:** EVs, Urine, miRNA, Physical exercise, Guanosine, Microscopy

## Abstract

Extracellular vesicles (EVs) enriched with bioactive molecules have gained considerable attention in nanotechnology because they are critical to intercellular communication while maintaining low immunological impact. Among biological matrices, urine has emerged as a noninvasive source of extracellular-contained liquid biopsy, currently of interest as a readout for physiological adaptations. Therefore, we aimed to evaluate chronic adaptations of endurance sport practice in terms of urinary EV parameters and evaluated by food consumption assessment. Two balanced groups of 13 inactive controls vs. triathlon athletes were enrolled; their urinary EVs were obtained by differential ultracentrifugation and analyzed by dynamic light scattering and transmission electron and atomic force microscopy. The cargo was analyzed by means of purine and miRNA content through HPLC-UV and qRT-PCR. Specific urinary EV signatures differentiated inactive versus endurance-trained in terms of peculiar shape. Particularly, a spheroid shape, smaller size, and lower roughness characterize EVs from triathletes. Metabolic and regulatory miRNAs often associated with skeletal muscle (i.e., miR378a-5p, miR27a-3p, miR133a, and miR206) also accounted for a differential signature. These miRNAs and guanosine in urinary EVs can be used as a readout for metabolic status along with the shape and roughness of EVs, novel informative parameters that are rarely considered. The network models allow scholars to entangle nutritional and exercise factors related to EVs’ miRNA and purine content to depict metabolic signatures. All in all, multiplex biophysical and molecular analyses of urinary EVs may serve as promising prospects for research in exercise physiology.

## Introduction

The extracellular fluids contain extracellular vesicles (EVs) that can be delivered to both parent cells and distant tissues. Both exosomes—deriving from the endocytic pathways—and ectosomes—generated at the level of the plasma membrane—can accumulate a plethora of molecules, navigate through the extracellular fluids, and fuse with the plasma membranes of the target cells thus influencing the functional activities, despite the heterogeneity of the two types of EVs [29].

Evidence supports the release of EVs in response to physical activity as muscle-derived EVs with paracrine and endocrine effects possibly resulting in health benefits [15] by mediating performance adaptations. Other tissues such as liver and adipose tissues account for factors released in response to exercise, which are collectively referred to as “exerkines”; exercise-induced exosomes (“exersomes”) and other EV types are currently thought to be a key path in intercellular communication during and after exercise [40]. Indeed, EVs released in response to physical exercise and packaged with molecules such as purines and miRNAs can exert autocrine, paracrine, and systemic effects to distant muscles and tissues. Therefore, the effects mediated by the intercellular communication of EV cargo delivered to recipient cells have opened novel intriguing fields of investigation into exercise physiology [46].

The small noncoding RNAs called miRNAs tune gene expression mainly by binding to the messenger RNA of protein-encoding genes. The complex regulatory network of RNA is linked to purine metabolism [53], and the endogenous pathways are linked to exogenous sources in a dynamic interconnection of miRNAs, purinergic system, and diet [26]. As diet does, physical exercise also affects both miRNAs and purine systems. Several purines increased their concentration in blood and urine after bouts of physical efforts [41], and the purines released into exosomes by muscles have been interestingly suggested as a path for muscle-brain crosstalk [35]. For what concern miRNAs, it has even been suggested that the disease-preventive molecular pattern of regular exercise is driven by exosomal miRNA modulation [17]. RNA is known to be prone to rapid degradation, but surrounded by membrane structures, miRNAs are protected from RNases and show a remarkable stability [47].

Within the field of EV research, urine has emerged as a noninvasive source of EV-contained liquid biopsy, useful as “diagnostic” biofluid for metabolic status as well as source for therapeutic molecules. Urine is therefore viewed as a dynamic bioactive fluid, whose alterations of EV composition represent valuable biomarkers and provide insights on renal pathophysiology. Indeed, to target urine as “diagnostic” biofluid for metabolic status is in accordance with the Extracellular RNA Communication Consortium that promotes investigation of several biofluids as source of markers as well as therapeutic molecules (https://exrna-atlas.org). The consortium developed an Atlas with 5309 exRNA-seq and ex-RNA qPCR sample profiles, primarily from serum, plasma, urine, saliva, and cerebrospinal fluid, collected across several different studies [33]. In the last years, research on urinary EVs (uEVs), in addition to a technical and medical focus, has moved to a physiological focus [43].

In spite of great efforts on downstream analyses of uEVs’ cargo, less attention has been devoted to the properties and topology of these enveloped structures. Instead, evaluation of the structural integrity and morphology of isolated EVs is needed both to proceed with the downstream molecular analysis of cargo and for comparing the morphology and integrity of EVs from different populations. Interestingly, growing body of evidence claims skeletal muscle as one of the largest secretory organ (considering fibers, satellite cells, endothelial cells, extracellular matrix, and immune cells) and one of the main EV producers in the human body, able to secrete in the bloodstream several myokines and exerkines packaged into EV and extremely useful for depicting metabolic status [18, 27]. A subset of circulating EVs, derived from exercised skeletal muscle, can enter the urine, both from perturbations of membrane-pore integrity, endothelial fenestrae of the glomerular filtration barrier, and transcytosis through podocytes. In addition, nonvesicular circulating molecules can be packaged into EVs and released into the urinary space after endocytosis by renal tubular cells [14]. Albeit, uEVs mainly originate from several cell types of the urogenital tract, residing immune cells and microorganisms; considering the large EV secretion by exercised body, the idea to find metabolic signature at urinary level is advocated and pursued because it can add value toward an accessible and scientific monitoring for healthy status. In this scenario, the analysis of nutrition profile and lifestyle- and exercise-related factors by means of the intriguing frontiers offered by the complex analysis and network models entailed novel data to understand these complex phenomena. We provided evidence for optimizing uEV research as a way of linking exercise physiology with metabolic status and prevent deleterious effects of inactivity

### Aim

We aimed to examine the shape and expression of metabolic/skeletal muscle miRNA along with the purine content of EVs extracted from the urine of long-term endurance-trained subjects (i.e., triathletes) and inactive subjects. Starting from the nutritional profile and through the complex analysis and network physiology approach, a uEV signature of the interactions between exercise-dependent and nutrition-related factors emerged.

## Materials and methods

The participants on voluntary base, 13 inactive male and 13 triathlon male athletes, were enrolled after presentation of the aim of the study in Abruzzo region Italy, according to protocol approved by University “G. d’Annunzio” Chieti-Pescara Committee board. They all were healthy young adults. Triathlon athletes did not train the day before the urine sampling.

### Participant characteristics

Participants compiled at home a specific questionnaire and self-reported values on height, weight, and waist circumference (WC). We calculated two indices of health that is the body mass index (BMI Kg/h^2^) and the WtHR (waist-to-height ratio), the latter calculated by dividing the WC by height, a useful screening tool for obesity and related cardiometabolic risks (cut-off 0.5) suitable for different sex and ethnic groups [6].

### Food consumption assessment

Food consumption was collected using diaries, structured into seven meals (three main meals and four snacks), and compiled by participants electronically. Specifically, each participant was instructed and self-compiled 3 diaries on nonconsecutive days (2 working days, 1 public holiday). The foods/recipes, drinks, and supplements taken were recorded, specifying their description (name, type, specific composition, cooking, taste, packaging, conservation, and fat used) and the quantity, using a photographic atlas. Food consumption was then entered in a specific web-based software database, Food Consumption Database (FOODCONS). This software was also used for coding and data processing in order to transform each registered food and recipe into weight in grams, amount of energy intake (EI), water, macronutrients (proteins, fats, saturated fatty acids (SFA), monounsaturated (MUFA) and polyunsaturated (PUFA), cholesterol, available, complex and soluble carbohydrates (CHO), fiber, alcohol), minerals such as calcium (Ca), phosphorus (P), magnesium (Mn), potassium (K), iron (Fe), and zinc (Zn), and vitamins such as vitamin C (Vit C), thiamin, riboflavin, niacin, vitamin B6 (Vit B6), vitamin B12 (Vit B12), vitamin D (Vit D), vitamin E (Vit E), vitamin K (Vit K), retinol, and vitamin A (expressed in equivalent of retinol or RE). The FOODCONS software and all related tools (databases on food composition, food diaries, and photographic atlas) were developed by the “Council for Agricultural Research and Analysis of Agricultural Economics” (CREA—Food and Nutrition) (https://www.crea.gov.it/en/web/alimenti-e-nutrizione) for use in food consumption survey in Italy and ad hoc adapted for this study.

### Small EV extraction from urines

**Fig. 1:**
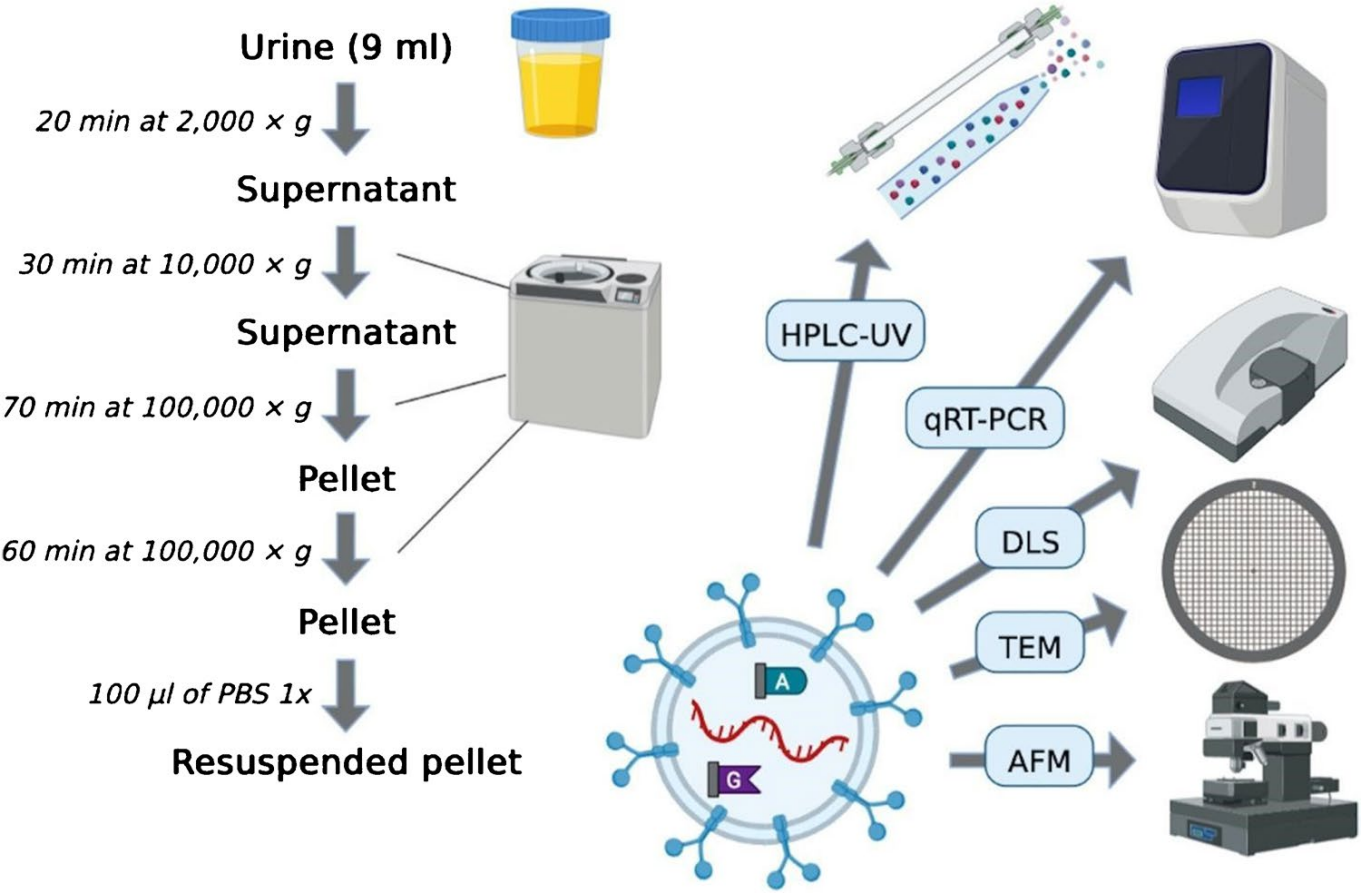
Schematic steps for EV extraction from urine

Urines from participants were collected in sterile containers as first morning specimens and stored at −80°C. Spot urine of each participant was used for urinalysis as a proxy of renal function, by quantifying creatinine, total proteins, and albumin and assessing osmolality. The whole study design is shown in Fig. 1. For the isolation and purification of small EVs from urine, the method of differential ultracentrifugation was carried out, with a first step at low speed, to remove cells and debris, followed by the subsequent consecutive isolation of large and small vesicles. In detail, after a first 10 min centrifugation at 300 × *g*, 4° C, 20 min centrifugation at 2000 × *g*, 4°C, followed to remove dead cells. The supernatant was then centrifuged 30 min at 10,000 × *g*, 4 °C. The resulting supernatant was ultracentrifuged for 70 min at 100,000 × *g*, 4 °C, to collect the pellet containing the small EVs. A final ultracentrifugation for 60 min at 100,000 × *g* was conducted to wash the small EVs, after resuspending in PBS 1X; the resulting pellet was resuspended in ≃100 μL of PBS 1X for later particle size, shape, and zeta potential analysis, along with molecular analyses. All centrifugation steps were performed with the Optima XL-100K ultracentrifuge, rotor SW 41 Ti Swinging-Bucket Rotor (Beckman Coulter, USA).

### Atomic force microscopy

The EVs were deposited on the graphene surface and scanned in the air by atomic force microscopy. Experimental data were obtained using the NT-MDT Solver Pro P-47 AF. The measurements were collected in semicontact mode by using a probe with a resonant frequency of 130 kHz and a spring constant of 4.4 N/m (HA_NC ETALON, NT-MDT). The EVs from scanning data were masked and marked as grains, to distinguish them from the background surface. The mask-covered area is considered to be area of interest for statistical analyses, and the quantities are expressed using integrals of the height distribution function with some powers of height. This postprocessing was done by Gwyddion, an open-source software for analysis of scanning probe microscopy measurements.

### Transmission electron microscopy

TEM analysis was performed with a JEOL JEM-1011 transmission electron microscope at 100 kV operating voltage, equipped with a 7.1 megapixel CCD camera (Orius SC1000, Gatan, Pleasanton, CA). Purified EV solution was simply drop-casted on 200-mesh copper TEM grids and dried in air.

### Dynamic light scattering and zeta potential

The average hydrodynamic diameter measurements were performed on a Zetasizer Nano instrument (Malvern, United Kingdom) equipped with a 10 mW He–Ne laser operating at 633 nm, fixed scattering angle of 173°. PBS-EV solutions were directly loaded in 1-cm polystyrene cuvette for DLS measurements, while they were diluted 6–10× before being loaded in Malvern DTS1070 cells for ZP measurements. Three measurements have been carried out for each sample.

### miRNA isolation and expression

Total miRNAs were isolated from uEVs using the miRNeasy Mini Kit (Qiagen) by adding the QIAzol Lysis Reagent (Qiagen®), following the manufacturer’s instructions. RNA concentration and quality were assessed with the Qubit™ microRNA Assay Kits and the Qubit RNA IQ Assay Kit, respectively, using a Qubit™ 4 Fluorometer (Thermo Fisher Scientific).

The extracted miRNAs were resuspended in 10 μL of UltraPure™ DEPC-Treated Water (Thermo Fisher Scientific). DNase treatment was not carried out because, as stated in the kit, the combination of QIAzol and RNeasy technologies is able to effectively remove most of the DNA and the analysis of mature miRNAs is not affected by the presence of minimal quantities of genomic DNA (Qiagen).

The polyadenylation of the miRNAs was carried out with the Poly(A) Polymerase (Diatech LabLine). Briefly, 0.10 μg of miRNA was incubated for 60 min at 37 ° C with a solution containing 10X Buffer, ATP (10 mM), and 2 U of enzyme, following the manufacturer’s guidelines (Takara Bio Inc.).

cDNA synthesis was performed using the OneScript® Hot Reverse Transcriptase (abm). Briefly, the reaction was developed in a final volume of 20 μL containing universal poly-T-adapter primer (2 μM; GCGAGCACAGAATTAATACGACTCACTATAGGT12VN; Eurofins Genomics), dNTPs, 5X RT Buffer, OneScript® Hot RTase, nuclease-free H_2_O, and polyadenylated miRNA. The tubes were incubated at 60° C for 30 min and 85° C for 5 minutes.

The miRNAs analyzed in this study (see Table 1) were chosen in that they are considered muscle-specific and likely involved in the regulation of energy metabolism during sports performance [2, 5, 10, 17]. Sequences of mature miRNA were derived from an online database (miRBase). The universal reverse primer (5^′^-GCGAGCACAGAATTAATACGAC-3^′^) in the amplification was fixed in all the reactions. Two microliter of the cDNA samples was added to the real-time PCR mixture consisting of primers (10 μM; Table 2) and BlasTaq™ 2X qPCR MasterMix (abm) in a final volume of 20 μL. Thermocycling for all reactions was for 3 min 95 °C, followed by 42 cycles of 15 s at 95 °C and 60 s at 60 °C. The cut-off value was set to 35 PCR cycles because below this value miRNAs are difficult to accurately compare [5].

**Table 1:** List of analyzed human miRNAs

hsa-miRNA	Sequences (5^′^-3^′^)	Accession
*miR-16-5p*	TAGCAGCACGTAAATATTGGCG	MIMAT0000069
*miR-486-5p*	TCCTGTACTGAGCTGCCCCGAG	MIMAT0002177
*miR-378a-5p*	CTCCTGACTCCAGGTCCTGTGT	MIMAT0000731
*miR-126-3p*	TCGTACCGTGAGTAATAATGCG	MIMAT0000445
*miR-27a-3p*	TTCACAGTGGCTAAGTTCCGC	MIMAT0000084
*let 7b-5p*	TGAGGTAGTAGGTTGTGTGGTT	MIMAT0000063
*miR-23a-3p*	ATCACATTGCCAGGGATTTCC	MIMAT0000078
*miR-133a-3p*	TTTGGTCCCCTTCAACCAGCTG	MIMAT0000427
*miR-133b*	TTTGGTCCCCTTCAACCAGCTA	MIMAT0000770
*miR-206*	TGGAATGTAAGGAAGTGTGTGG	MIMAT0000462
*miR-34a-5p*	TGGCAGTGTCTTAGCTGGTTGT	MIMAT0000255
*miR-92a-3p*	TATTGCACTTGTCCCGGCCTGT	MIMAT0000092

**Table 2:** Anthropometrics and training data of IN and TR subjects

	Age (years)	Hour*p*Week (h)	Weight (kg)	Height (cm)	Wc (cm)	BMI (kg/m^2^)	WtHR
IN (*n* = 13)	42 ± 3.4	1.4 ± 1.4	88.5 ± 11.3	178 ± 6.8	100 ± 9.2	27.3 ± 2.6	0.56 ± 0.05
TR (*n* = 13)	47 ± 8.8	10 ± 5.8	77.0 ± 8.3	179 ± 6.7	85 ± 6.0	24.1 ± 1.8	0.48 ± 0.05

The data are reported as mean ± standard deviation

*IN* inactive, *TR* triathletes, *n* number of enrolled participants, *HourpWeek* weakly hours spent training, *Wc* waist circumference, *BMI* body mass index, *WtHR* waist-to-height ratio

Expression levels of miRNA in triathletes were normalized with respect to the housekeeping miR-16-5p [23] and were expressed with respect to the inactive group.

### miRNA target sequence computational analysis

Specific miRNA target genes were identified using mirPath v.3 (http://snf-515788.vm.okeanos.grnet.gr/), an online software that uses data from the TarBase v7.0 database, setting the *p* value threshold to 0.05. Target genes were then visualized within specific pathways available in the Kyoto Encyclopedia of Genes and Genomes (KEGG) database.

### Purine analysis by HPLC

The HPLC analyses for adenosine series were carried out in isocratic conditions with phosphate buffer (40 mM, pH=5.8) as solvent A and MeOH as solvent B (90:10, A:B). ODS stationary phase (Xtimate C18, 250 × 4.6 mm, 5 μm) column was used. The total run time was 25 minutes. All compounds were detected at their maximum wavelength of 264 nm.

The HPLC analyses for guanosine and derivates were carried out using phosphate buffer (40 mM, pH=7.0) as solvent A and acetonitrile as solvent B in gradient conditions. ODS stationary phase (Xtimate C18, 250 × 4.6 mm, 5 μm) column was used. Each compound was observed at 256 nm, except for guanosine that was detected at 259 nm. Stock solution for each chemical standard was obtained at 1 mg/mL in water and then diluted in MilliQ water to obtain working solutions (from 0.2 to 10 μg/mL). All samples were directly analyzed (dry EVs suspended in 44 mL of MilliQ water) without further purification steps.

The method for the adenine series shows linearities up to 10 μg/mL and shows limit of quantification of 0.2 μg/mL for ADP, AMP, ADE, and cAMP, while 0.5 μg/mL for ATP (based on signal-to-noise ratio of 10 and BIAS% values). The limits of detections were 0.08 mg/mL for ADP, AMP, ADE, and cAMP, while 0.15 μg/mL for ATP (based on signal-to-noise ratio of 3). The method for guanosine series shows linearities up to 5 μg/mL and shows limit of quantification of 0.1 μg/mL (based on signal-to-noise ratio of 10 and BIAS% values). The limit of detections was 0.03 μg/mL (based on signal-to-noise ratio of 3).

They matched perfectly all the anthropometric parameters.

### Statistical analyses

Statistical analysis was performed on Jamovi software (version 2.3.18.0) and Prism Version 9 (GraphPad Software, San Diego, USA). Before proceeding with the comparisons, the Shapiro-Wilk test for normality and the Levene test for homogeneity of variances were evaluated.

A series of t-test for independent samples was then conducted with Welch’s method, calculating the *p* values, the Hedges’ *g* as effect size, and the 95% confidence interval, integrating the results with the creation of violin plots, thanks to the JJStatsPlot module implemented on the software. If assumptions were violated, a Mann-Whitney test was conducted. Comparisons were then corrected for BMI, waist-to-height ratio, or age, through ANCOVA analysis, calculating the *p* values and the partial *η*^2^ as effect size. Roughness parameters were analyzed using unpaired t-test. Spearman’s correlation matrix for further analysis of weighted undirected networks was built as static correlation graphs: edges were shown for rho over 0.24 and *α* < 0.05. Nodes were positioned with the circular layout algorithm; the greater the weight of correlation, the greater the size of the edge. Recalling the formula of combination, *N* ! /[*K* ! (*N* − *K*)!], with *N* number of nodes, and subsets of *K* = 2 (an undirected link between 2 nodes) elements, a maximum set of $\frac{N\left(N-1\right)}{2}$ edges is possible; setting *E* as the number of observed edges, the edge density was then calculated as 2*E*/[*N*(*N* − 1)]. Other network metrics considered were weighted degree, as the absolute sum of weights of a node to its “neighbors,” and betweenness centrality, which ranks important a node if many paths between nodes in different groups must pass through it.

## Results

The participants have been grouped into inactive (IN) and trained (TR) according to their engagement in weekly training, about 1 versus 10 hours, respectively. The TR group was composed exclusively by triathletes used to perform endurance training on a regular basis. Self-reported anthropometrics and training data and related indices are shown in Table 2.

The IN group was overweight (BMI 27.3 ± 2.6) with a WtHR slightly higher than the cut-off (0.56 ± 0.05), while the TR group was on average into healthy ranges (BMI 24.1 ± 1.8 and WtHR 0.48 ± 0.05) also considering that they spent weakly 10 ± 5.8 hours training. Considering the type of training of triathletes, a weekly energy expenditure as metabolic equivalent of task (MET) minutes per week of their sports activity was calculated; their training was considered moderate-to-vigorous activity, i.e., 6 MET [1], and their energy expenditure ranged from 720 to 5940 MET minutes per week.

### Nutritional profile and macro and micronutrient intake

Food diaries were compiled by 15 IN and 8 TR people. We excluded from our analysis people who followed a dietary plan for weight loss (1 IN subject). All participants consumed main meals (breakfast, lunch, and dinner); one IN subject never consumed snacks.

The nutritional profile revealed an increased energy intake in breakfast, dinner, and snacks in triathletes, who had a higher percentage intake of lipid in breakfast and snacks and in contrast a lower percentage of protein and carbohydrate at lunch, with respect to inactive controls (Table 3).

**Table 3:** Percentage of energy, water, and macronutrient intake (%) between main meals and snacks in the group of inactive controls and triathletes, as obtained by food diaries

		Energy intake	Water^	Proteins	Lipids	Available carbohydrates
Breakfast	*IN*	11%	10%	10%	9%	17%
	*TR*	17%	12%	14%	15%	22%
Lunch	*IN*	48%	37%	50%	51%	38%
	*TR*	28%	27%	26%	28%	28%
Dinner	*IN*	37%	35%	38%	37%	38%
	*TR*	40%	44%	48%	40%	37%
Snacks	*IN*	4%	18%	2%	3%	7%
	*TR*	15%	17%	12%	17%	13%

*IN* inactive control group, *TR* triathlon group

^Water drunk and taken by food

In addition to the expected greater intake of water in triathletes (*p* = 0.017, *g* = 1.315), it is interesting to observe the differences in the distribution of water intake between the meals. Triathletes introduce water especially at dinner as opposed to inactive who introduce water in similar percentages between lunch and dinner. The intake of water in snacks was also different, with a percentage of 25% of the total daily water intake for athletes. Differences also existed in macro- and micronutrient intake (Table 4). Triathletes overall introduced more calories than sedentary people (3160 ± 889 and 2505 ± 688 kcals, respectively; *p* = 0.098, *g* = 0.820).

**Table 4:** Data of the energy and nutrient intakes for the two groups expressed as mean ± SD and percentage, according to nutritional guidelines for moderate/high-intensity training (Triathlon Guide Line Tr GL^*1,2,3^) and recommended daily allowances for Italian population (It DRVs^*4^)

*Daily intake*	IN (*n* = 14)		TR (*n* = 8)		Tr GL^*1,2,3*^	It DRVs^*4*^
	*Mean*	SD*	*Mean*	SD*		
Energy intake_EI (MJ)	10	3	13	4		
Energy intake_EI (kcal)	2505	688	3160	888.6	2000–7000^(1)^	
Water (g/day)^	2264.3	565.2	3158.3	792.7	4000–5000^(2)^	2500^(AI)^
Protein (g/day)	103.4	27.2	131.8	31.9	ni	63^(PRI)^
Protein (g/kg bw/day)	1.2	0.2	1.8	0.6	1.2–2.0^(1–2)^	0.9^(PRI)^
Fat (g/day)	114.3	39.5	133.5	56	ni	ni
SFA (g/day)	33.9	12	38.5	19.3	ni	ni
PUFA (g/day)	15.3	5.7	22	9.3	ni	ni
Cholesterol (mg/day)	410.3	167.6	424.6	236.8	ni	<300^(SDT)^
Available carbohydrates (g/day)	234.7	76.7	372.9	175.5	ni	ni
Available carbohydrates (g/kg bw/day)	2.7	0.8	5.2	3	8^(1)^–12^(2)^	ni
Simple carbohydrate (g)	59.7	19.1	103	50.5	ni	ni
Dietary fiber (g/1000 kcal/d)	7.4	2.6	10.4	3.9	ni	12.6–16.7^(RI)^
Alcohol (g/day)	25.7	25.2	3.3	6.9	0	0
*% total energy from*	IN (*n* = 14)		TR (*n* = 8)		Tr GL^*1,2,3*^	It DRVs^*4*^
	*Mean*	SD*	*Mean*	SD*		
Proteins (% EI)	16	4	17	4	ni	10–15^(RI)^
Lipid (%EI)	41	14	38	16	Up to 50^(1)^	≤30^(RI)^
SFA (%EI)	12	4	11	5	ni	<10^(SDT)^
PUFA (%EI)	5	2	6	3	pDRVs°	5–10^(RI)^
Available carbohydrates (%EI)	35	11	44	21	ni	45–60^(RI)^
Simple carbohydrates (%EI)	9	3	12	3	ni	<15^(SDT)^
*Minerals*	IN (*n* = 13)		TR (*n* = 8)		Tr GL^*1,2,3*^	It DRVs^*4*^
	*Mean*	SD*	*Mean*	SD*		
Potassium (mg/day)	3484.6	1165.6	4107.9	995	pDRVs°	3900^(AI)^
Phosphorus (mg/day)	1558.5	420.2	2058.6	669.6	pDRVs°	700^(PRI)^
Calcium (mg/day)	749.9	323.7	1291.4	642.8	pDRVs°	1000^(PRI)^
Magnesium (mg/day)	421.7	290.6	560.9	227.6	pDRVs°	240^(PRI)^
Iron (mg/day)	16.9	6.9	23	8.4	Up to 70% of requirement	10^(PRI)^
Zinc (mg/day)	16.2	5.5	32	39.5	pDRVs°	12^(PRI)^
*Vitamins*	IN (*n* = 13)		TR (*n* = 8)		Tr GL^*1,2,3*^	It DRVs^*4*^
	*Mean*	SD*	*Mean*	SD*		
Thiamine (mg/day)	1.3	0.4	4.8	6.8	pDRVs°	1.2^(PRI)^
Riboflavin (mg/day)	1.8	0.7	5.1	6.9	pDRVs°	1.6^(PRI)^
Niacin (mg/day)	26.6	7.8	40.4	17.2	pDRVs°	18^(PRI)^
Vitamin C (mg/day)	118.1	91.8	125.9	83.5	pDRVs°	105^(PRI)^
Vitamin B6 (mg/day)	2.5	0.8	4.6	3.2	pDRVs°	1.3^(PRI)^
Vitamin A (REs μg/day)^§^	717.1	1696.8	410.2	240	pDRVs°	700^(PRI)^
β-Carotene (μg/day)	2762.9	1923	4664.8	3665.3	ni	ni
Vitamin K (μg /day)	308.9	367.1	621.8	1022.6	pDRVs°	140^(AI)^
Vitamin E (mg/day)	18.5	9.9	29.8	17	pDRVs°	13^(AI)^
Vitamin D (μg/day)	2.8	1.6	10.6	9.1	pDRVs°	15^(AI)^
Vitamin B12 (μg/day)	12.4	15.4	14.5	12.5	pDRVs°	2.4^(PRI)^

*IN* inactive control group, *TR* triathlon group, *SD* standard deviation, *ni* not indicated, *bw* body weight, *AI* adequate intake, *AR* average requirement, *PRI* population recommended intake, *RI* reference intake range for nutrients, *SDT* suggested dietary targets

^Water drunk and taken by food

°Dietary reference values for nutrient intake for the general population [42]

^**§**^Vitamin A expressed as retinol equivalents (REs): 1 retinol equivalent (RE) = 1 μg retinol = 6 μg β-carotene

([42]; [7]; [44]; [21])

The calories introduced by the IN group derived mainly from lipids and saturated fats, while in triathletes from proteins, polyunsaturated fatty acids, and available carbohydrates. The daily carbohydrate intake in the TR group was almost significantly higher than that in control (*p* = 0.051, *g* = 1.273) close to the specific recommendations for the type of sport [7], which recommend for 1–2 hours of training per day from 5 to 7 grams of carbohydrates per kg of body weight. The TR protein intake per kg of body weight was greater than control (*p* = 0.026, *g* = 1.481) and differed slightly from the recommendations for the general population (1.4–1.6 grams of protein per kg of body weight). The TR fat intake seemed higher than control in absolute values, but in percentage of energy intake was similar (38% vs. 41% on average); it is important to remember that the amount of fat consumed daily with food must not be below 20% of total caloric energy in order not to compromise the intake of fat-soluble vitamins, carotenoids, and essential fatty acids including omega-3 [19].

The amount of TR total fiber was almost significantly higher than that introduced by IN (*p* = 0.079, *g* = 0.926). The IN daily intake of water, fiber, calcium, and vitamin D was lower, while that of cholesterol is higher, than DRVs. In particular, calcium intake of IN was almost significantly lower than TR (*p* = 0.053, *g* = 1.129). The TR and IN daily intake of vitamin D was lower, while that of cholesterol was higher, than DRVs. The alcohol content derived mainly from beer that IN was used to drink more that TR (126±185 vs. 83±115 g/day). For what concerns purine-rich foods (g), IN were used to eat more meat (181±137 vs. 107±101 g/day) and less fish (92±60 vs. 119±103 g/day) and legumes (17±32 vs. 33±32 g/day) than TR (Table 5). The large variance in purine-rich food intake across participants limited the possibility to run statistical comparisons.

**Table 5:** Intake of purine-rich foods in TR vs. IN groups

	Beer (g)	Meat (g)	Fish (g)	Legumes (g)	MAI
IN	126±185	181±137	92±60	17±32	2.7±1.5
TR	83±115	107±101	119±103	33±32	3.6±2.2

Data are reported as mean value ± standard deviation

*MAI* Mediterranean adequacy index

Adequacy to the Mediterranean diet (MD), assessed by MAI (Mediterranean adequacy index), was very low in both groups (and nonsignificantly different between them, *p* = 0.326, *g* = 0.487) because below the acceptable value 5 [12]; the score considers the amount of kcal from bread, pasta, vegetables, fruits, cereals, legumes, potatoes, fish, nut, olive oil, and red wine, divided by kcal from milk, cheese, meat, eggs, animal fats, drinks, sweets, and sugar, and values over 5 were considered acceptable [4].

### Urinalysis

**Fig. 2:**
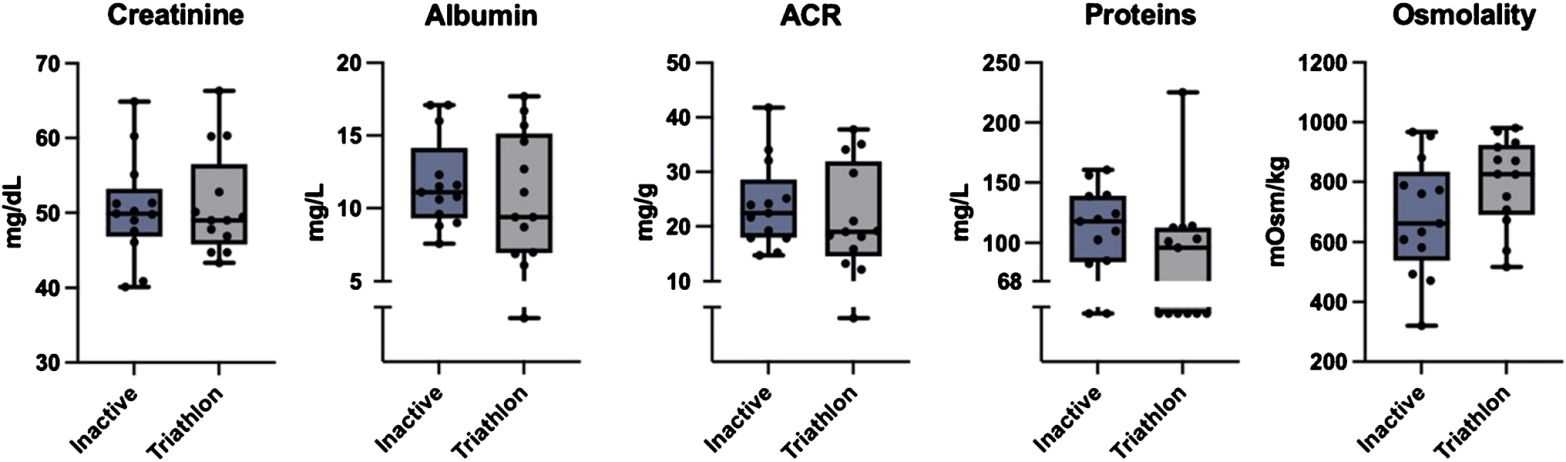
Results of urinalysis. ACR: albumin-to-creatinine ratio. One point of albumin and 8 points of protein data were under the detection level of 5 mg/L and 68 mg/L, respectively

As shown in Fig. 2, inactive controls and triathletes did not differ in terms of both creatinine (*p* = 0.814, *g* = 0.090) and albumin (*p* = 0.532, *g* = 0.241). Instead, protein concentration was generally lower and osmolality was slightly and not significantly higher (*p* = 0.098, *g* = 0.656) in triathletes.

### Urinary EV shape and size distribution

**Fig. 3:**
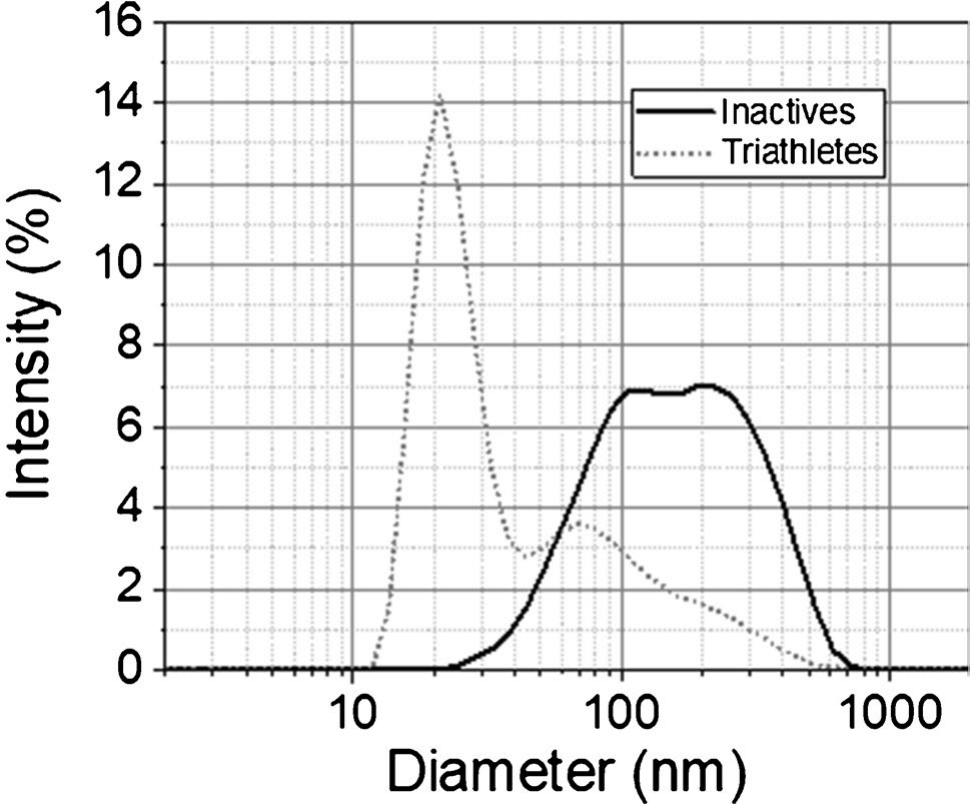
EV average size distribution: the figure reports the comparison between the average size distributions of extracellular vesicles collected by IN controls and TR. In both cases, a broad distribution has been detected by DLS measurements, with a significant difference in the distribution profile. Large vesicles (>100 nm of diameter) were predominant for IN people, where two peaks of comparable intensity can be observed at ~100 nm and ~200 nm. Instead, small vesicles (<40 nm of diameter) dominated the distribution for TR, with a tail extended above 100 nm

In order to have a deeper insight on the shape and on the shape distribution of EVs, a wide morphological analysis has been carried out by means of dynamic light scattering (DLS), transmission electron microscopy (TEM), and atomic force microscopy (AFM). While DLS allows to easily acquire information on the particle size distribution measured on a large number of macrovesicles (see Fig. 3), AFM and TEM led the light on the size and the shape of a reduced number of small EVs, likely exosomes and very small ectosomes. For what concerns zeta potential, similar results were obtained for inactive controls and triathletes, confirming previous insights on a potential of about −25 mV. Such moderate values can be ascribed to the high conductivity of the medium (~2.5 mS/cm), due to an elevated PBS concentration. Indeed, in such conditions, EVs are surrounded by a large number of ions which interact with their surface, altering their electrical double layer, thus lowering their repulsive forces and zeta potential.

**Fig. 4:**
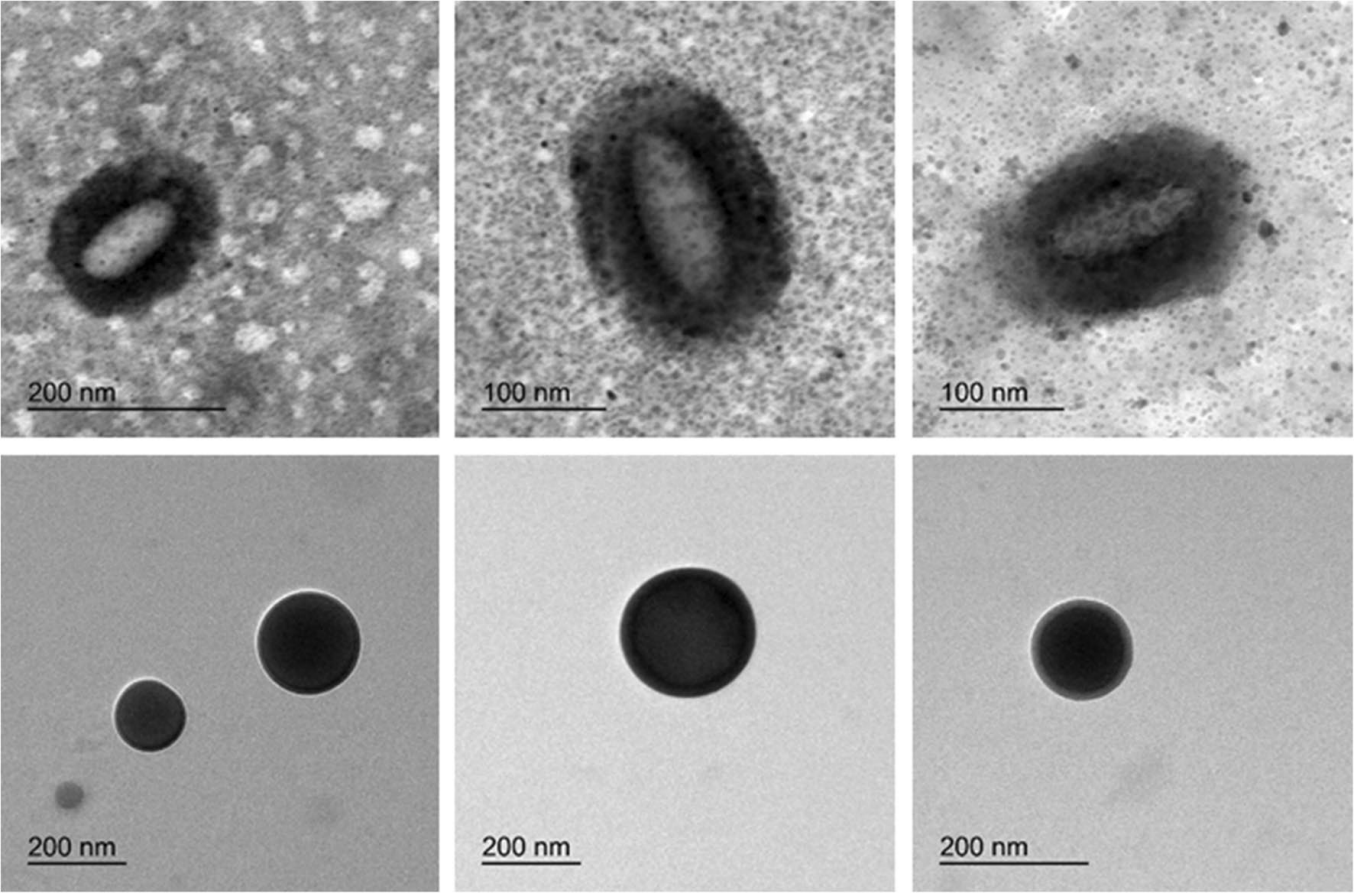
EV shape morphology: TEM analysis showed different shapes of EVs comparing IN (upper panels) to TR (lower panels). The upper panel reports three images of EVs collected by inactive people, while the lower panel reports three images of EVs collected by triathletes. The number of analyzed macrovesicles is very low due to the low EV concentration of the solution used for the grid preparation. In addition, with any treatment aimed to increase the contrast of biologic materials, such as exosomes, only large vesicles can be detected

The morphology and size of some EVs have been investigated by TEM (Fig. 4). EVs collected by TR appeared sphere-shaped with various sizes from 50 to 200 nm; EVs collected by IN have a spheroid-like shape with an aspect ratio of 2. Such results were compatible with the size distributions measured by DLS.

**Fig. 5:**
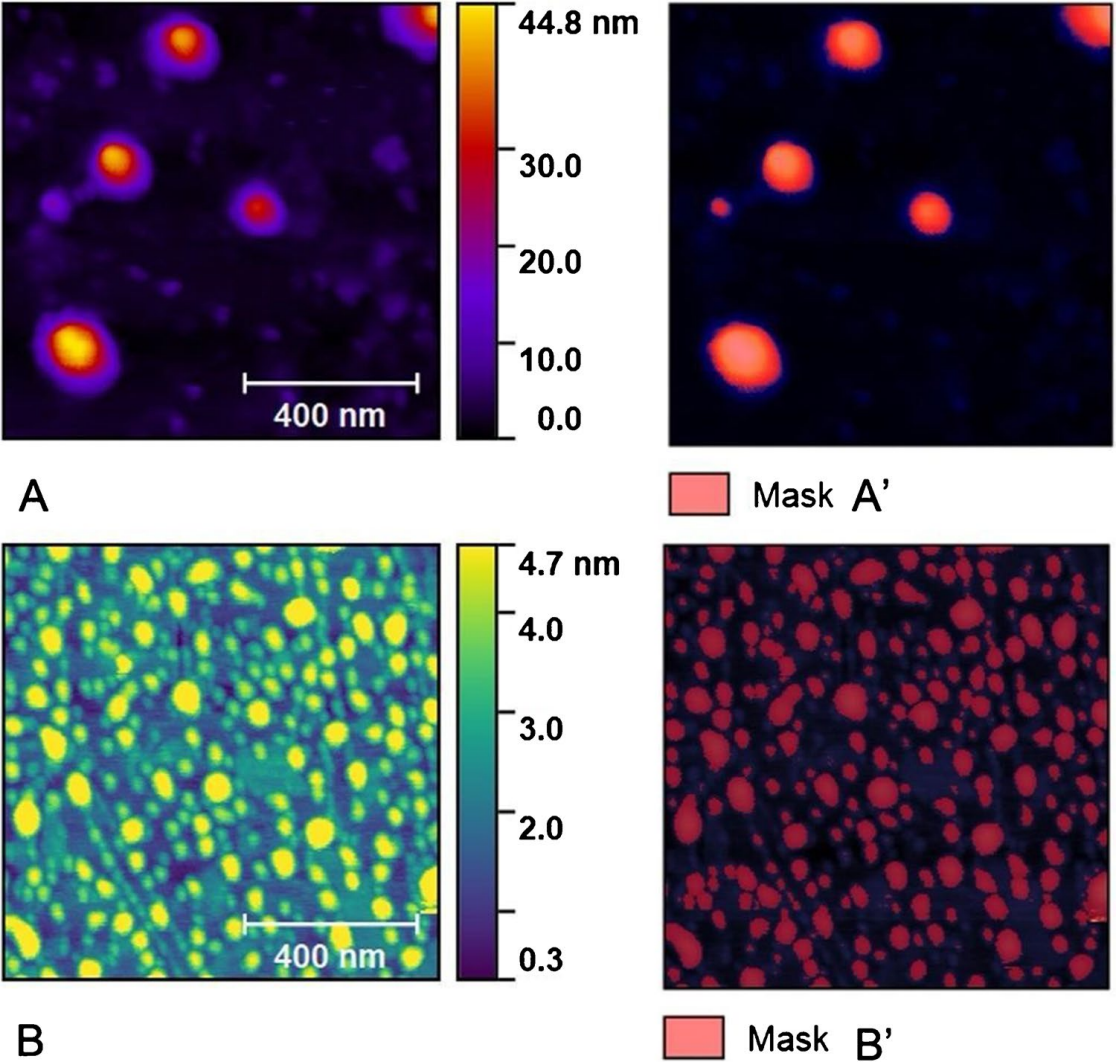
The images derived from AFM scanning and are representative of EV topography of IN (**A** and **A**’) and TR (**B** and **B**’). Panels **A** and **B** represent the original scanning area while **A**’ and **B**’ represent scanning after applying the mask to isolate vesicles on which analysis of roughness have been performed and reported in Table 6

The shape analysis of EVs was conducted also by means of AFM that revealed larger EV diameter of IN compared to TR (Fig. 5). The RMS roughness is the mean square of height irregularities *S*_q_ that is computed from the 2nd central moment of data values, also known as variance of height distribution. The “grain-wise RMS” on the other hand determines the mean value for each grain separately, and the variance is then calculated from these per-grain mean values. Finally yet importantly is the mean roughness or *S*_a_ value of height irregularities. Mean roughness (*p* < 0.001, *g* = 2.897), grain-wise RMS (*p* < 0.001, *g* = 3.008), and root mean square roughness (*p* < 0.001, *g* = 2.727) as computed by AFM were greater in EVs from IN with respect to TR (Table 6). Moreover, the number of EV we found in AFM scanning area was different between IN and TR, 13±9 vs. 155±53, respectively.

**Table 6:** The roughness parameters obtained by AFM scanning on masked EVs

	Average value	RMS roughness (*S*_q_)	RMS (grain-wise)	Mean roughness (Sa)
IN (*n* = 8)	4.9±1.8×10^−3^	20.4±8.4×10^−3^	17.6±6.6×10^−3^	16.9±6.6×10^−3^
TR (*n* = 6)	4.3±0.4×10^−3^	1.7±0.3×10^−3^	1.4±0.2×10^−3^	1.3±0.1×10^−3^

Data are reported as mean value ± standard deviation (*n* = 8 for IN and *n* = 6 for TR)

### Urinary EV metabolic miRNA content

**Fig. 6:**
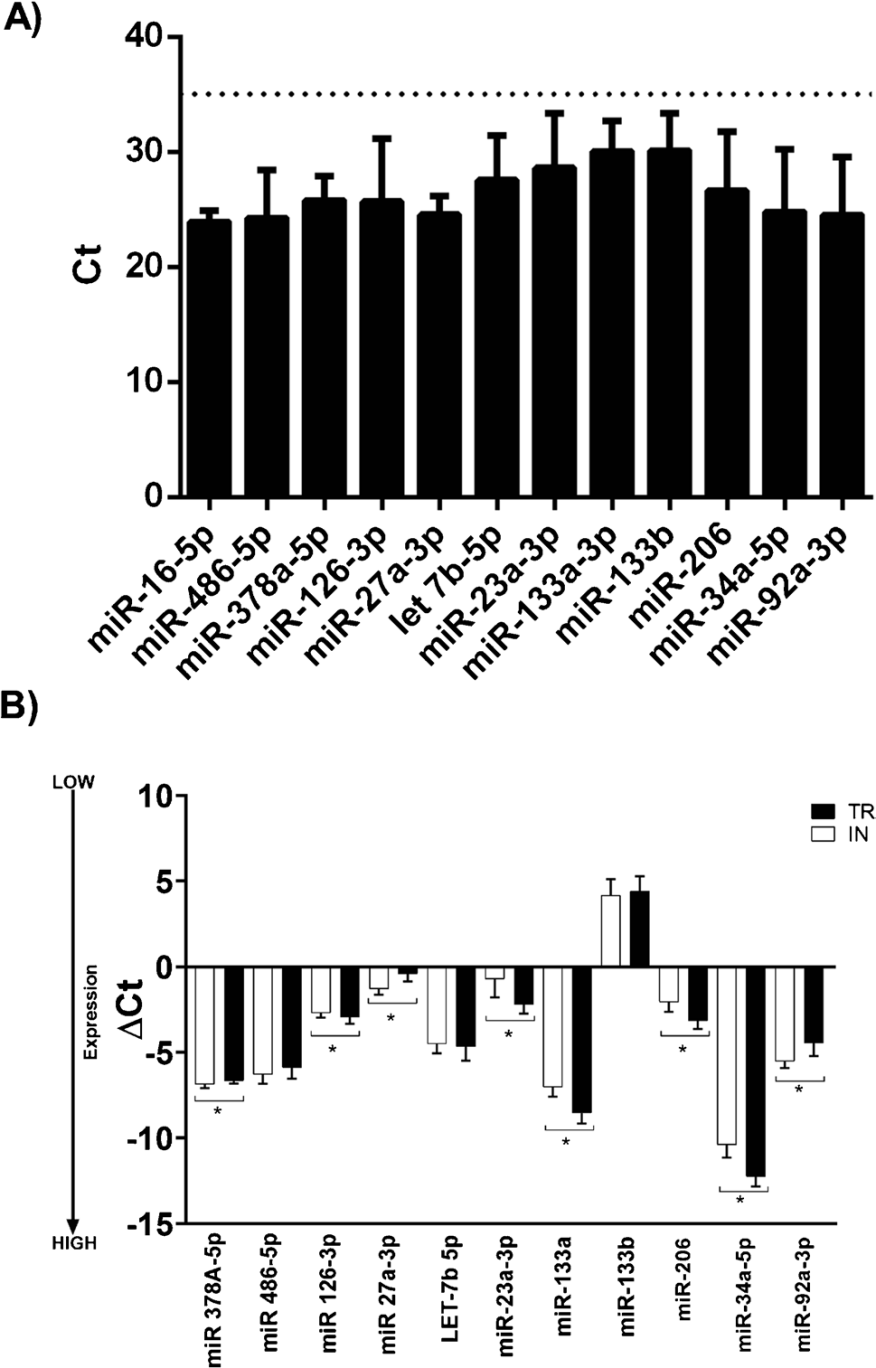
Levels of muscle-specific miRNAs in uEVs. miRNA levels are expressed as mean ± standard deviation (SD) of threshold cycle (Ct) values obtained from 28 subjects (**A**). DeltaCt (ΔCt) values of microRNAs differentially expressed in EVs between IN males and TR male athletes (**B**). Bars represent mean ± SD values of ΔCt per sample category. Asterisks denote differential expression *p* values: (*) <0.05

Results on expression profiles of EV miRNAs are shown in Fig. 6. Data were calculated based on reference miRNAs according to a threshold cycle (Ct) value (Fig. 6A). Since the Ct cut-off value for low-copy miRNAs is typically 35 [30], we judged that all analyzed miRNAs can be quantified and their expression changes compared in response to physical exercise.

**Fig. 7:**
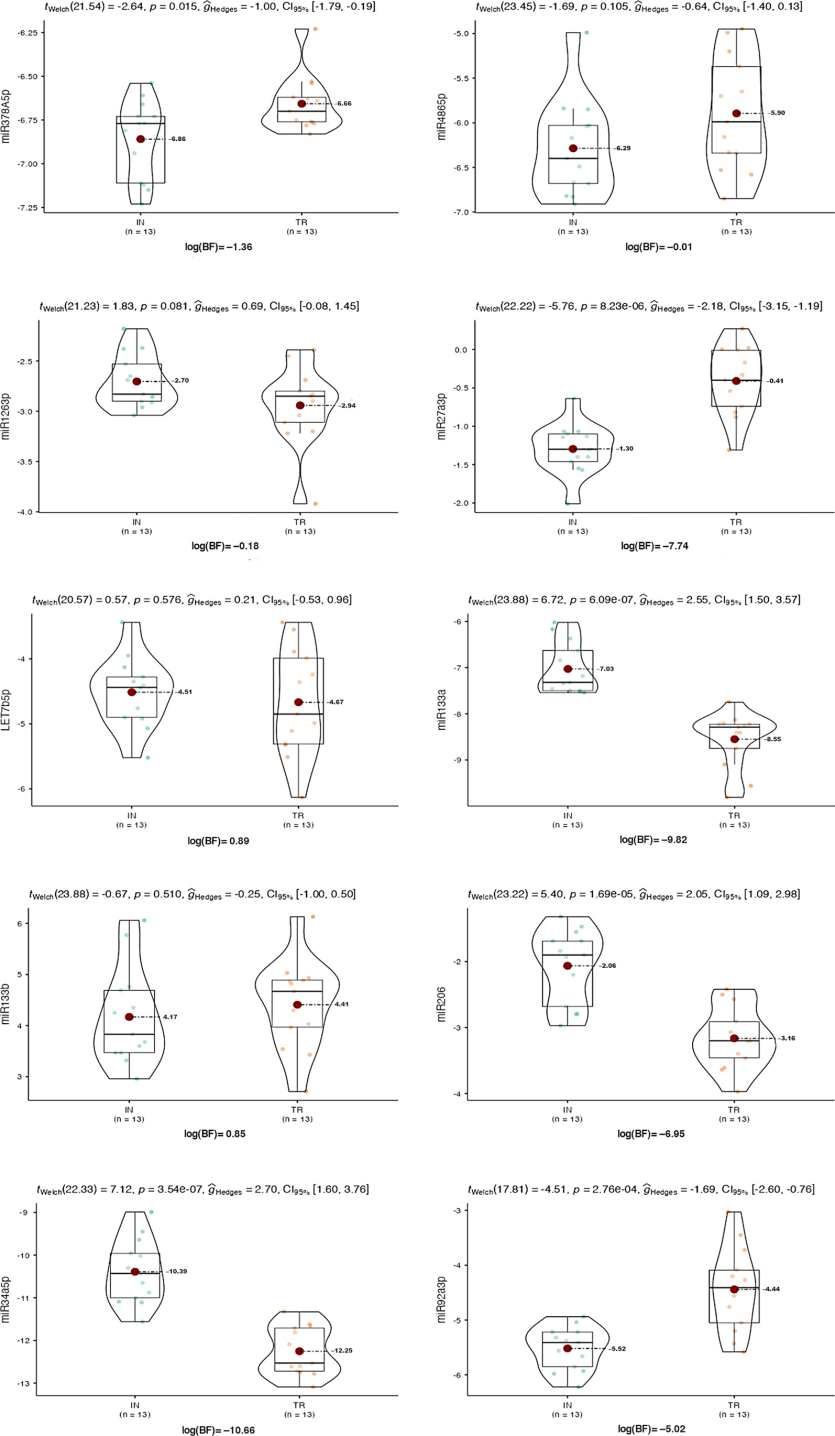
Muscle-specific miRNA expression: the violin plots show the median, mean (big dots), IQR, and all data points of 10 miRNAs analyzed (miR23a-3p has not been analyzed with this method due to the violation of assumptions of normality and homogeneity). The scale factor for the Bayesian test has been set as *r* = 0.707, assuming a Cauchy prior distribution; Bayes factors (BF) are reported on a logarithmic scale as log_e_(BF_01_).

Comparison of the EV miRNA expression profiles between IN and TR groups is shown in Figs. 6B and 7. We found eight miRNAs with significant altered expression between the two groups. After correcting for age, BMI, and WtHR, these differences remained substantially unchanged (Fig. 6). The miR378a-5p ΔCt was significantly lower for IN (*p* = 0.014, *η*^2^_p_ = 0.225); this difference was mitigated by correcting for the WtHR (*p* = 0.129, *η*^2^_p_ = 0.098). Similarly, mir486-5p showed a trend towards significance with lower ΔCt for IN (*p* = 0.104, *η*^2^_p_ = 0.106); this difference disappeared by correcting both for WtHR (*p* = 0.673, *η*^2^_p_ = 0.008) and for BMI (*p* = 0.860, *η*^2^_p_ = 0.001). There was a trend towards significance for miR126-3p, with higher ΔCt for TR (*p* = 0.080, *η*^2^_p_ = 0.123); this difference was mitigated by correcting both for WtHR (*p* = 0.123, *η*^2^_p_ = 0.100) and BMI (*p* = 0.203, *η*^2^_p_ = 0.069). The most marked difference in terms of expression was found for miR27a-3p, with lower ΔCt for IN (*p* < 0.001, *η*^2^_p_ = 0.580). This difference still remains evident when correcting for BMI (*p* < 0.001, *η*^2^_p_ = 0.454) or WtHR (*p* < 0.001, *η*^2^_p_ = 0.465). No difference was found for mirLet7b-5p (*p* = 0.575, *η*^2^_p_ = 0.013); however, strong trends emerged after correcting for both WtHR (*p* = 0.070, *η*^2^_p_ = 0.136) and BMI (*p* = 0.080, *η*^2^_p_ = 0.127). It is worth mentioning that miR-206, often associated by skeletal muscles, was detectable in urine EVs, confirming the idea that uEV pool also contains nanovesicles that originated from distant tissues. Among the results, miR-92a-3p ΔCt was significantly lower for IN than for TR group (*p* < 0.001, *η*^2^_p_ = 0.459); this difference was partially mitigated after correcting both WtHR (*p* = 0.002, *η*^2^_p_ = 0.357) but less for BMI (*p* < 0.001, *η*^2^_p_ = 0.414). No differences emerged for miR-133b ΔCt. Instead, miR-23a-3p ΔCt was significantly lower for TR than for IN group (*p* < 0.001, *r* = 0.858). Similarly, miR-133a ΔCt was lower for TR than for IN (*p* < 0.001, *η*^2^_p_ = 0.653); this difference remained after correcting both for WtHR (*p* < 0.001, *η*^2^_p_ = 0.474) and for BMI (*p* < 0.001, *η*^2^_p_ = 0.562). The miR-206 ΔCt was also significantly lower for TR than for IN group (*p* < 0.001, *η*^2^_p_ = 0.548); this difference was partially mitigated after correcting for WtHR (*p* = 0.003, *η*^2^_p_ = 0.326) and BMI (*p* = 0.003, *η*^2^_p_ = 0.329). Finally, miR-34a-5p was significantly lower for TR than for IN group (*p* < 0.001, *η*^2^_p_ = 0.679), and the differences remained after correcting for WtHR (*p* < 0.001, *η*^2^_p_ = 0.590) and BMI (*p* < 0.001, *η*^2^_p_ = 0.536).

Identification of cellular targets and functions associated with miRNAs whose expression was significantly different between groups (*i.e*., miR-92a-3p, miR-27a-3p, miR-23a-3p, miR-133a, miR-206, and miR-34a-5p) is displayed in Table 7. Biological processes most significantly (*p* < 0.000001) affected by differentially expressed miRNAs were specific hallmarks of cell growth, including postmitotic cell growth and intercellular adhesions but also DNA damage, oxidative stress, and apoptosis. Notably, both the p53 signaling pathway (hsa04115) and the Hippo signaling pathway (hsa04390) were affected by all six miRNAs, while miR-133a was not directly implicated in the regulation of cell cycle (hsa04100).

**Table 7:** List of the top pathways, with the number of corresponding genes, associated with the microRNAs significantly differentially expressed between IN and TR groups. Pathways were identified from DIANA-miRPath v3.0 web server

Selected KEGG pathways	*p* value	#Genes	#miRNAs
Cell cycle (hsa04110)	5.01e-10	86	5
p53 signaling pathway (hsa04115)	5.77*e* − 08	52	6
Hippo signaling pathway (hsa04390)	9.75*e*−08	82	6
Adherens junction (hsa04520)	1.62*e*−07	47	5
Protein processing in endoplasmic reticulum (hsa04141)	7.21*e*−06	96	5
Endocytosis (hsa04144)	7.71*e*−06	115	6
TGF-β signaling pathway (hsa04350)	1.01*e*−05	51	6
Lysine degradation (hsa00310)	1.20*e*−04	28	5
Thyroid hormone signaling pathway (hsa04919)	3.34*e*−04	72	5
FoxO signaling pathway (hsa04068)	8.00*e*−04	76	5
Estrogen signaling pathway (hsa04915)	1.42*e*−03	53	5
Erbβ signaling pathway (hsa04012)	1.94*e*−03	55	6
Regulation of actin cytoskeleton (hsa04810)	3.38*e*−03	107	6
Fatty acid metabolism (hsa01212)	4.20*e*−03	23	5
Focal adhesion (hsa04510)	6.19*e*−03	107	5
AMPK signaling pathway (hsa04152)	1.10*e*−02	66	6
MAPK signaling pathway (hsa04010)	1.64*e*−02	122	6
Phosphatidylinositol signaling system (hsa04070)	1.65*e*−02	43	6

### Purine content in urinary EVs

In this work, we demonstrated for the first time that GUA was the only purine detectable in urine EVs and was present in about 50% of tested uEVs while Ado and ATP in all samples are below limit of quantification and the other purines sparsely represented (see Table 8). GUA concentration was of in the order of hundreds nM with no significant difference comparing IN *vs.* TR. In this study, the applied HPLC method allows the accurate (precise and true) identification and quantification of the purine content by mean of a simple, fast, rugged, validated instrument configuration. The main goal is also related to the use of isocratic elution profile that allows an easy method transfer to other instrumentation, maintaining the chromatographic performances, as also highlighted in a previously published paper [36].

**Table 8:** Concentration of purines packaged into uEVs (data are mean±SD)

	Guanosine-based (μg/mL)				Adenosine-based (μg/mL)				
	GTP	GDP	GMP	GUA	ATP	ADP	AMP	ADE	cAMP
IN1				0.20 (±0.02)	BLQ		BLD		
IN2				0.44 (±0.03)	0.87 (±0.07)			BLQ	
IN3					BLD				BLD
IN4				0.35 (±0.03)	BLQ				
IN5									
IN6					BLD				
IN7		BLQ		0.45 (±0.04)	BLQ			BLQ	
IN8				0.22 (±0.01)	BLQ				
IN9									
IN10					BLQ				
IN11							BLD		
IN12				0.16 (±0.01)	BLQ				
IN13				0.17 (±0.01)					
TR1					BLQ		BLQ	BLQ	
TR2					BLQ				BLQ
TR3				0.90 (±0.07)	BLQ				0.28 (±0.02)
TR4					BLQ				
TR5				0.39 (±0.04)				BLQ	BLQ
TR6				0.15 (±0.01)			BLD		
TR7				0.28 (±0.03)	BLQ				
TR8					0.50 (±0.03)				
TR0					BLD				
TR10				0.64 (±0.06)					
TR11	BLQ						BLD		
TR12	0.21 (±0.02)	BLD			BLD				
TR13				0.63 (±0.5)					

*BLD* below limit of detection (0.03 μg/mL), *BLQ* below limit of quantification (0.1 μg/mL)

### Network analysis

**Fig. 8:**
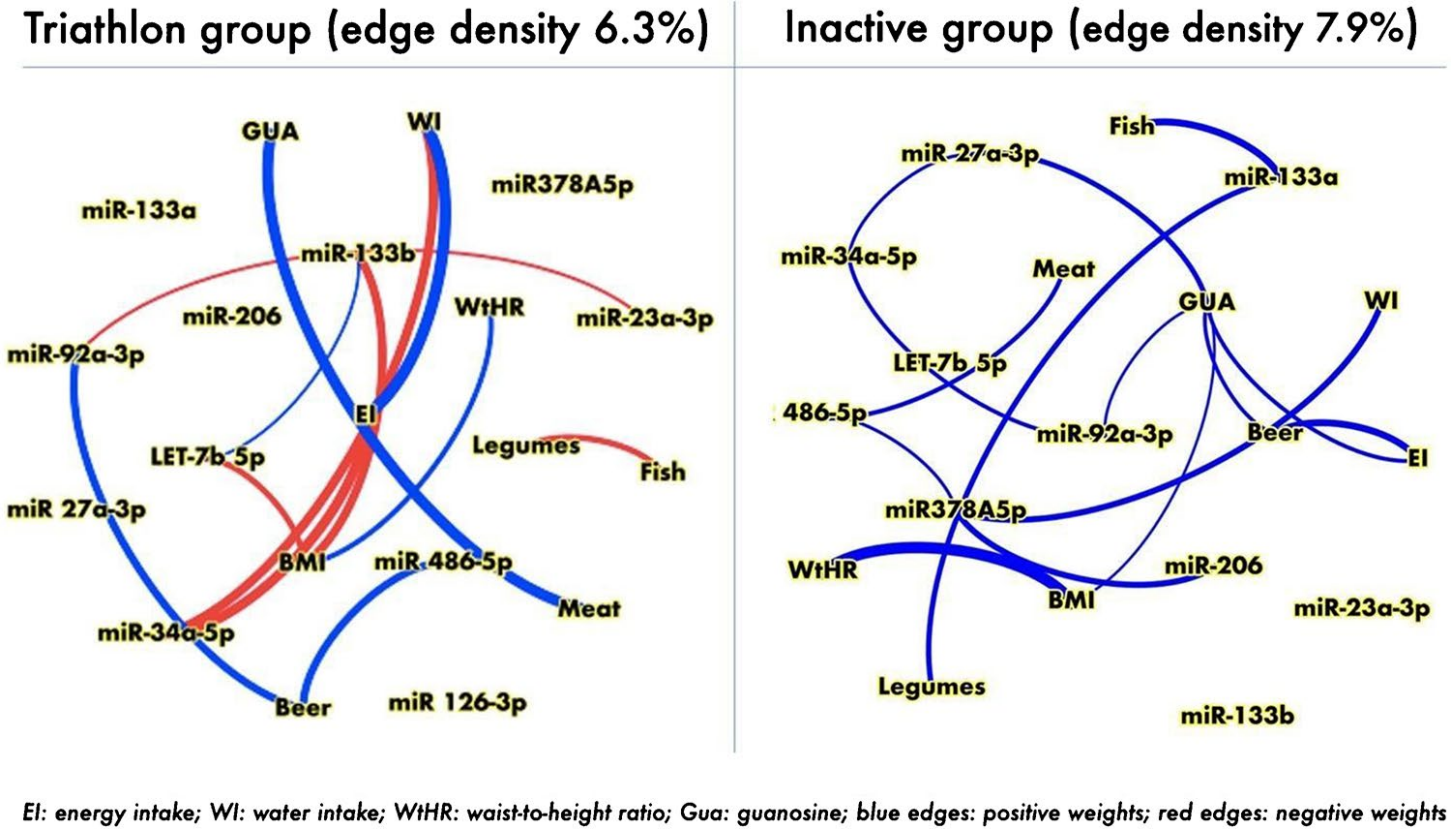
Network analysis: the figure shows the network analysis connecting food intake, anthropometric data, purines, and miRNA content of uEVs in the TR (triathlon) and IN (control) groups. It should be noted that in the triathlon group there were less edges and that some of them were related to negative correlations among variables

The complex analysis of data by means of network analysis revealed that several links across domains (anthropometrics, food intake, miRNAs, and purines) were present (Fig. 8) and they were differently associated in the IN and TR groups. Considering the number of nodes and observations and the number of edges above the threshold, it was not possible to robustly compute and rank nodes basing on centrality measures. It is worth mentioning that the connections between BMI-WtHR-LET7b-5p present in TR was absent in IN. Moreover, miRNA 27a-3p and 378-5p were disconnected in TR while connected to GUA and meat in the IN group. The miRNA126-3p was disconnected in both groups.

Guanosine was linked to meat in TR, but not in IN where it resulted connected to miRNA27a-3p and beer.

## Discussion

Physical inactivity can negatively affect many anthropometric parameters such as weight, abdominal fat accumulation, and body mass index and can affect the expression of miRNA involved in significant biological mechanisms. Food intake also provides insights into how athletes develop a more thoughtful and personalized approach to nutrition, which allows them to train better, recover, and adapt optimally, while avoiding the onset of illness and injury.

Our data suggest that athletes pay more attention to the caloric breakdown into macronutrients, in the various meals, presumably with the aim of achieving better physical performance, especially during the training phase. Supporting the right energy needs is, in fact, the main nutritional goal in endurance athletes where the availability of energy must not be less than 30–45 kcal/kg/day, with carbohydrates from 3–5 g/kg/day for low-intensity training up to 8–12 g/kg/day in more intensive training periods. By evaluating the intake of liquids, in particular water, a greater income in triathletes was found, in support of the need for reintegration during the training and competition phases. Despite the achievement of the macronutrient requirement by athletes, both groups show a very low MAI index, which suggest a diet based on food like milk, cheese, meat, and eggs that does not fit with MD model. Meat, fish, legumes, and beer are the most purine reach food, and guanosine often represents >60% of the total purine content [52]. Considering that several factors can affect real purine content, such as region and seasonal food production, food processing (e.g., dehydration), cooking, and storage [20, 52], we cannot accurately quantify purine intake in our groups and then differentiate dietary from endogenous contribution to the body purine pool. However, we know diet is an exogenous source of nucleotides mostly as nucleosides adenosine monophosphate (AMP) and guanine monophosphate (GMP) [54]. Dietary purines are absorbed through the small intestine and, following the purine metabolic pathway, their metabolites are degraded and excreted as uric acid, the latter’s serum and urine concentration mostly affected by adenine and hypoxanthine [20, 52]. Future direction should take into consideration serum level of uric acid and investigate dietary contribution to the guanosine level. We cannot know if food- or beer-derived purines are also adsorbed packaged into EV and in urine we found part of them; however, the link between miRNA 27a-3p and GUA found in uEVs of the IN group linked to EI and beer deserves further analysis considering the possible role of mitochondria in this node.

The link between physical exercise and EVs has been extensively studied, and EVs are currently interpreted as a key path to drive the tissue crosstalk and possibly the multisystemic benefits [34]. Since urine can be a reservoir of EVs coming from the circulation after crossing the glomerular filtration barrier [9], their characterization represents an intriguing as a noninvasive liquid biopsy tool for detecting the exercise-induced metabolic adaptation. In our work, confirming previous results [51], ultracentrifugation method yielded intact globular EVs; however, the expected single Gaussian distribution was found only for the TR group (Fig. 2). In addition, TR uEVs were smaller, with a lower roughness and an evident spheroid-like shape, rather than EVs from the IN group. Suggestively, we can say that uEV shape of IN with respect to endurance-trained subjects is peculiar: a likeness of “wrinkled sofa” versus a “spherical ball,” respectively. Moreover, the EV morphological properties such as membrane diameter and roughness are parameters to be taken into consideration for the viscous friction force that they create during their transit both at contact with endothelium of vessels and during filtration. This friction feature has been poorly considered, and it deserves to be further characterized for EV properties. Only under speculative point of view, it could induce a senescence phenotype in human microvascular endothelial cells, could increase stiffness, and could give alteration to the EV adhesion properties. For this purpose, AFM has been previously used to characterize nanomechanical properties that can serve to distinguish signature between different cell types [22]. Previously, it was shown that surface membrane proteins and lipid layer contribute to form membrane ruffles that influence the cell body roughness, but rarely this investigation has been conducted for EVs, despite the topographic mapping can be extremely informative also for EV properties. Moreover, the AFM scanning allowed to visualize another interesting aspect that is the EV numerosity, at a glance one order of magnitude more in TR group with respect to the IN group. However, this aspect deserves a dedicated investigation.

Our results, in terms of both biophysical and molecular characterization of uEVs, were supposed to be mostly unrelated to any dysfunction of renal filtration barrier, since urinalysis did not reveal differences in both creatinine and albumin level, nor in the ACR index. The greater osmolality found in triathletes can be interpreted as a sign of dehydrated urines. In any case, both molecular concentrations and osmolality remained in physiological levels in all participants.

Special attention has been paid to miRNA presence since the investigation of uEVs’ miRNA content could be useful to understand the possible EV derivation. Specifically, we aimed to have evidence that urine, a simple and noninvasive biological fluid, can help in profiling a long-term endurance exercise habit or help in distinguishing inactive behavior. The first encouraging result is that all investigated miRNAs were found in uEVs, supporting their possible derivation from distant tissues such as skeletal muscle. Notably, miR-206 is associated with tissues not included in the urinary tract (among them skeletal muscle) and its presence in uEVs further testifies the enrichment of EV pool in EVs of distant tissue derivation. Speculatively, the results show the potential for using EVs as tools to investigate the molecular mechanisms of skeletal muscle adaptation to exercise. Indeed, with the exception of miR-92a-3p and miR-27a-3p, which were found to be downregulated in TR, four other miRNAs (*i.e.*, miR-23a-3p, miR-133a, miR-206, and miR-34a-5p) were upregulated in TR with respect to the IN group. Age, BMI, and WtHR had only slight, if any, influence on the comparison between the two groups. Therefore, our findings indicate that the expression of these EV miRNAs is altered as consequence of long-term sport engagement, as for moderate-to-vigorous activity or about 6 MET.

It is well known that increased mitochondrial biogenesis, function, and hypertrophy of skeletal muscle are important adaptive responses to regular exercise. In this regard, the upregulation of miR-133a, miR-206, miR-34a-5p, and miR-23a-3p reflects previous data suggesting that the expression of these miRNAs can increase after athletic performance. Mooren and colleagues [32] reported a significant increase in miR133a and miR-206 levels after a marathon run. The highest increase was observed for miR-206, whose expression enhanced nearly 20-fold with respect to pre-exercise levels. Interestingly, these levels remained high for more than 24 h after the marathon. In addition, the authors also found that both miR-133a and miR-206 correlated to maximum oxygen uptake V̇O_2max_ and running speed at individual anaerobic lactate threshold (VIAS). These latter findings, together with our results, contribute to demonstrate that the skeletal muscle-related miR-133a and miR-206 are relevant for aerobic performance. In addition, the combined increase of miR-133 and miR-206 can enhance not only myogenic differentiation but also myoblast proliferation and self-renewal. Regarding miR-34a-5p, de Gonzalo-Calvo and colleagues [11] demonstrated that the expression of this miRNA showed a sudden peak immediately after a marathon race and returned to baseline levels within 24 h. In addition, significant miR-34a-5p increases aligned with ROS levels in an ischemia/reperfusion mouse model suggesting a potential role of this miRNA in regulating SIRT1-dependent ROS reduction [45]. Finally, previous studies demonstrated an increase also in miR-23a-3p expression levels following resistance exercises [10, 38].

In the present study, we also observed downregulation of EV miR-92a-3p and miR-27a-3p in TR compared to IN individuals. As reported by Wen and colleagues ([50], p. 3), the reduction in circulating miR-92a-3p may be induced by resistance exercise intervention that leads to a decreased oxidative stress response. This mechanism seems to be related to a reduction of miR-92a-3p-mediated repression of HO-1 (HMOX1), an antioxidant enzyme found in vascular endothelial cells and smooth muscle cells. In this context, considering the decreased EV roughness found in the TR group, we can hypothesize a link between miR-92a-3p downregulation and the positive effects on EV lipid metabolism possibly mediated by inducible HO-1 enzyme and its circulating enzymatic reaction products during to the long-term exercise [39].

With reference to miRNA 27a-3p expression, the decrease observed in TRs compared to INs can be supported by a recent study linking aerobic resistance training to sensitivity of human lymphocytes [2]. The authors found that lymphocytes of endurance athletes were characterized by a downregulation of the proapoptotic miR-27a. This result is possibly due to the need for maintaining lymphocyte homeostasis and trigger resistance mechanisms to effectively cope with exercise-induced cellular stress factors. In addition, overexpression of miR-27a-3p was found to induce glycogen accumulation in mouse myoblasts leading to mitochondrial fragmentation, utilization of lipids, and reduced breakdown of glycogen [8]. Therefore, we speculated that downregulation of miR-27a-3p observed in TRs might counteract mitochondrial degradation and accumulation of glycogen in the muscles promoting the processes of glycolysis and cellular respiration. The differentially expressed miRNAs were predicted to target several pathways, particularly cell cycle, p53, and Hippo signaling pathways. These findings significantly extend the evidence supporting these miRNAs as potential biomarkers of physical activity and exercise training. In this regard, the Hippo pathway is considered a critical mediator of adult skeletal muscle fiber growth and atrophy [49].

Given the possibility of miRNAs packaged into EVs to contribute to cell-cell communication and exert biological function to recipient cells has been questioned [3], further studies are needed to clarify if systemic EVs carrying miRNAs, possibly reflected in the pool of uEVs, should be seen only as a molecular fingerprint, rather than possible predictors of molecular cascades of target tissues. Into this view, despite skeletal muscle can be considered as the largest secretory organ, with a continuous and massive release of EVs, this organ contributes only in a small percentage for circulating EVs, at least in animal models [16]. It has been suggested that the most skeletal muscle-derived EVs reside within the skeletal muscle microenvironment and express a specific miRNA signature that regulates muscle biology, at least in animal models [48].

Back to uEV cargo found in this study, in our opinion, the presence of purine into the uEVs is also very interesting, not only considering the well-defined role of adenosine-based molecules in both the cellular energetic supply and extracellular pleiotropic factors, but mainly for the role that guanosine-based molecules could have in the crosstalk between skeletal muscle and metabolic status. Specifically, it has been demonstrated that both different neuronal and glial cell types along with satellite cells of the skeletal muscle release GUA and GTP [24, 36]. In particular, GUA exerts positive neurotrophic effect [13]. Considering that exercise has been recognized having a positive role on mood [25], recently it has been proposed that GUA packaged into exosomes released by skeletal muscle cells could be delivered to the brain as mediator of exercise-dependent beneficial effects [35]. As argued above, a subset of circulating EVs can enter the urine, by putative mechanisms of transient perturbations of membrane-pore integrity, endothelial fenestrae of the glomerular filtration barrier, transcytosis through podocytes and nonvesicular circulating molecules can also be found into uEVs by packaging and release into urine after endocytosis by renal tubular cells [14]. Although uEVs mostly originate from several cell types of the urogenital tract, it can be therefore speculated that in nonpathological conditions uEVs can reflect, at least in part, the systemic status.

In this work, we demonstrated for the first time that guanosine is present in uEVs at micromolar concentration while adenosine in all samples is below the limit of quantification. Despite we cannot distinguish the guanosine source as exogenous or endogenous, specifically from food intake and/or the cellular production, this result opens new perspectives for exercise-dependent EV guanosine role in tissue crosstalk. In particular, the link between miRNA 27a-3p and GUA in uEVs of inactive people deserves further investigation as marker of sedentary status, while the only link with meat intake in triathletes suggests GUA delivery and utilization by metabolic active body.

The interesting approach of network analysis highlights the importance of complex analysis in complex phenomenon. The increased edge density of IN with respect to the TR group indicates more complicated interactions among factors *vs.* a clean connection of two or three factors in the TR group, as the strong relation between miRLet7b, WtHR, and BMI. Moreover, the connection between miR27a-3p and GUA in the IN group could support the negative role of both factors in mitochondrial dynamics, in particular, a possible negative influence on GUA-linked molecule production and a possible negative influence of miR27a-3p mitochondrial fragmentation on the IN group.

In this vein, we could also suggest future investigation of the possible link between GUA and miR-92a-3p regulation along with their involvement in HO-1/Nrf2 and MAPK/ERK signaling pathways ([37], p. 3), highlighting the relevance of these molecular targets in metabolic status.

### Limitations

Since low sample size may have biased the results, larger sample studies are needed for obtaining robust inferential insights. Size and surface topography are affected by the EV isolation method [51], raising issues in comparisons between different studies. Caution is also required in comparing zeta potential, as PBS concentration affects the results, i.e., it shows a shift toward less negative values in the presence of less diluted (higher PBS concentration) samples [31]. Specific factors to distinguish and sort exosomes and ectosomes [28] were not implemented, masking the type-specific effects of EVs, despite this was not the aim of the current study. The sorting of different populations would also help in depicting, if any, difference in roughness and zeta potential. Confirmation of EV core proteins (such as tetraspanins CD9 and CD63) should be designed in further studies. Despite isolation procedures and sample preparation were comparable, the differences in TEM images, e.g., concerning the background, should be furtherly investigated. An additional isolation method will be required in further studies to confirm purine and miRNAs as uEV cargo rather than just co-elution results. The heterogeneity of energy expenditure within each group may have biased the results. Further studies may involve larger sample size, focusing on MET minutes a week to discriminate inactive (e.g., <500 MET minutes a week) people, and professional sports practice to define highly adapted athletes.

## Conclusions

Endurance sportsmen and inactive people have a differential signature of uEVs, both for distribution, shape, roughness, and content in biomolecules. Intriguingly, a clear difference has been observed from microscopy imaging, with a spheroid shape and smaller size of uEVs from the triathletes while increased roughness and size for inactive people.

As far as content is concerned, the substantial difference between inactive people and triathletes for miR-92a-3p, miR-27a-3p, miR-23a-3p, miR-133a, miR-206, and miR-34a-5p does not appear markedly influenced by the variables of BMI, WtHR, and age, thus suggesting that the metabolic pathways regulated by these miRNAs are closely related to physical activity and training level. Therefore, future research involving larger samples of inactive people and triathletes will complement the compelling evidence shown by this work. We can underline how the benefits associated with endurance training imply the understanding of multisystem adaptations and the identification of complex signaling networks, a focal point for the implementation of interventions aimed mainly at the prevention and treatment of dysmetabolic diseases, such as diabetes and obesity.

As perspectives, studying uEVs and their content can be used as a meaningful, noninvasive approach in sports science and exercise physiology. A deeper urinalysis accounting for several biophysical and molecular characterization could help in depicting the specific role of some variables such as pH and hydration status in EV release while offering several opportunities for normalizing parameters. A specific set of markers derived from multiplex analyses can be implemented. Sorting small EVs to depict the differential cargo of exosomes and ectosomes will specify the population-specific trafficking. Roughness from AFM deserves further attention to depict its possible relevance in EVs signaling. Moreover, network approaches on larger sample size could unveil original interconnections and emerging factors, likely adding novel insights for defining long-term adaptations of humans in response to physical activity.

The likelihood of uEVs in exercise physiology can rely on the possibility to depict the exercise-specific EV adaptations in a noninvasive biofluid; moreover, given participants are not affected by urinary tract diseases, EV isolates from urine should contain fewer contaminants (such as protein aggregates, lipoproteins, and viruses) than those from blood thereby representing a valid and meaningful source of biomarkers. All in all, while there remain important challenges (i.e., the isolation and tracking of tissue-specific EVs) for the field, our work is in line with the exciting prospect of developing a deeper and more holistic understanding of how exercise is able to promote systemic adaptations.
